# Infectious Mononucleosis-Induced Splenic Infarction: Perhaps More Common in Healthy Individuals Than Previously Thought

**DOI:** 10.7759/cureus.39472

**Published:** 2023-05-25

**Authors:** Gabriella Mamo, Stephanie Erickson, Karthikram Komanduri, Dewid Zayas, Niti Aggarwal

**Affiliations:** 1 Internal Medicine, HCA Florida Ocala Hospital, Ocala, USA; 2 Internal Medicine, University of Central Florida College of Medicine, Orlando, USA

**Keywords:** internal medicine, ebv, coagulopathy, infectious disease, infarction, monospot, splenic, spleen, epstein-barr virus, infectious mononucleosis

## Abstract

Infectious mononucleosis (IM) is a clinical syndrome that presents as a triad of fever, pharyngitis, and lymphadenopathy. In most cases, it is caused by the Epstein-Barr virus (EBV), which spreads through upper respiratory secretions, particularly saliva, earning its name as the *Kissing Disease*. In most cases, IM is self-limiting and resolves in two to four weeks without significant sequelae following supportive care. Although rare, IM has been associated with several serious and sometimes life-threatening complications, involving almost any organ system. Splenic infarction is one rare complication of IM due to EBV infection. In the past, IM-induced splenic infarction in the setting of EBV was believed to be rare and mostly limited to patients with underlying hematologic comorbidities. However, we propose this condition to be more common and more likely to occur in individuals without significant medical history than previously suspected. We report a relatively healthy young male patient in his thirties, with no previous history of coagulopathy or complex medical conditions, who was found to have IM-induced splenic infarction.

## Introduction

Infectious mononucleosis (IM) is a clinical syndrome that presents as a triad of fever, pharyngitis, and lymphadenopathy. It is most often caused by the Epstein-Barr virus (EBV), or human herpesvirus 4, in approximately 90% of cases. It occurs most commonly in young, fertile individuals aged between 15 and 25 years. The virus spread through upper respiratory secretions, particularly saliva. Thus, it has garnered itself the colloquial name of the *Kissing Disease* [1,2].

The diagnosis of suspected IM can be confirmed by performing a heterophile antibody (mononucleosis spot or *Monospot*) test. This test is usually positive in the first three weeks of the infection but then begins to rapidly decrease. However, this test has low sensitivity, resulting in up to 25% false negatives. In approximately 10% of adults, and even more commonly in children, the heterophile antibody does not develop. These IM-like infections are most often caused by cytomegalovirus (CMV). In cases in which the heterophile antibody test is negative but IM is still suspected, an EBV Antibody Profile may be performed [3]. This assay tests for four components in the serum: EBV viral capsid antigen (VCA) IgG, VCA IgM, EBV early antigen (EA), and EBV nuclear antigen (EBNA). EBV VCA IgM, EBV VCA IgG, and EBV EA are present in the acute phase of an EBV infection. VCA IgM and EA decrease as the disease progresses, while VCA IgG persists and EBNA rises. Positive VCA IgM, VCA IgG, and/or EA indicate primary acute infection. Positive VCA IgG and/or EBNA indicate past infection. EBV PCR and/or EBV viral load measurement can also be used when serological tests are inconclusive [4]. Laboratory analysis in the setting of IM also frequently reveals lymphocytosis and/or elevation of atypical lymphocytes with or without absolute leukocytosis. Liver enzymes may also be transiently elevated in 50% to 80% of patients [4,5].

Common signs of IM on physical exam include palatal petechiae, splenomegaly, and hepatomegaly [4,5]. In most cases, IM is self-limiting and resolves in two to four weeks without significant sequelae following supportive care. However, a minority of individuals will develop complications of IM that can involve almost any organ system [2]. Some of these complications include pneumonia, myocarditis, pericarditis, anemia, thrombocytopenia, pancreatitis, glomerulonephritis, parotitis, and various neurologic disorders, all of which occur in 1% or less of cases. While rare, these complications can be serious and even life-threatening [2,4].

An even rare and lesser-studied complication of IM is splenic infarction. Splenic infarction can be caused by many different mechanisms: thromboembolic disease (e.g., atrial fibrillation, infective endocarditis, and valvular disease), autoimmune disease (e.g., systemic lupus erythematosus and antiphospholipid syndrome), viral illnesses (e.g., EBV, CMV, and VZV), blood-borne malignancies, myeloproliferative disorders, hemoglobinopathies (e.g., sickle cell anemia and hereditary spherocytosis), hereditary thrombophilia (e.g., protein C or S deficiency and antithrombin III deficiency), blunt abdominal traumas, inflammatory processes (e.g., pancreatitis), and various other causes of hypercoagulability (e.g., polycythemia vera, exogenous estrogen use, and malignancy) [6,7].

Previous retrospective studies have reported the most common presentation of splenic infarctions to be abdominal pain, which occurs in 80% to 84% of patients [7]. Other patients may experience left-sided pleuritic chest pain, caused by abdominal pain radiating to the left hemithorax, known as Kehr’s sign [8]. Most patients with splenic infarctions have elevated lactate dehydrogenase (LDH) in laboratory studies [5]. However, this finding is nonsensitive and nonspecific and, therefore, should only raise suspicion for splenic infarction when present.

In the past, splenic infarctions in the setting of EBV-induced IM were believed to be rare and mostly limited to patients with underlying hematologic comorbidities. However, we propose this condition to be more common and more likely to occur in individuals without significant medical history than previously suspected.

## Case presentation

We report a 32-year-old male patient with a past medical history of obesity, depression, post-traumatic stress disorder, and migraines who presented to the emergency department with a one-week history of myalgias, generalized fatigue, and progressively worsening sharp left upper quadrant (LUQ) pain. The pain radiated to the left shoulder and worsened with inhalation. The patient also reported a 101 °F fever two days prior that resolved with acetaminophen and diarrhea for the previous three days. He denied sore throat, chest pain, nausea, and vomiting.

The patient denied tobacco or intravenous drug use. He denied any recent sick contacts or travel. He was sexually active with one partner. Besides his obesity, he was otherwise healthy, with no previous history of any coagulopathies or complex medical conditions. Family history was significant for a mother with Evan's syndrome, a rare autoimmune disorder leading to thrombocytopenia and hemolytic anemia, as well as multiple deep vein thrombosis (DVT) and pulmonary emboli.

Vital signs on admission included a temperature of 98.4 °F, blood pressure of 186/106 mmHg, heart rate of 98 beats/minute, respiratory rate of 16 breaths/minute, and SpO2 of 98% on room air. On physical examination, the patient was found to be obese, with a body mass index (BMI) of 54.9. He was in mild distress secondary to pain. He had mild discomfort with deep palpation over the LUQ, but the abdomen was soft and nondistended without guarding or rebound tenderness. No pharyngitis or lymphadenopathy was noted. The remainder of the physical examination was unremarkable.

Complete blood count revealed mild polycythemia (hemoglobin 17 g/dL), no significant leukocytosis (white blood cell count 4,800 mm^3^), and normal platelet count (185,000 mm^3^). Liver function tests revealed elevated aspartate transaminase (AST) 144 U/L, alanine transaminase (ALT) 147 U/L, alkaline phosphatase (ALP) 156 U/L, and LDH 1,420 U/L. Bilirubin was within normal range (1 mg/dL). Other normal routine laboratory findings included creatinine 1 mg/dL, blood urea nitrogen 15 mg/dL, and lactic acid 1.1 mmol/L. The erythrocyte sedimentation rate was 26 mm/hour, and the C-reactive protein level was 7.5 mg/L. Coagulopathy results showed a prothrombin time of 12.8 seconds and an activated partial thromboplastin time of 30.2 seconds. D-dimer was elevated at 2,506 ng/mL fibrinogen equivalent units (FEUs).

**Figure 1:**
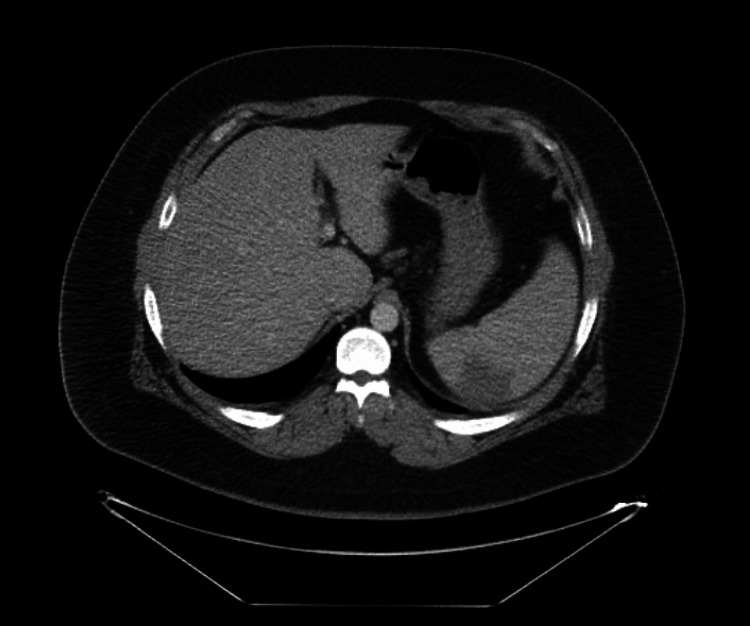
CT abdomen and pelvis with IV contrast (axial view). Triple-phase abdominal CT reveals wedge-shaped hypodensity at the posterior and medial aspects of the spleen. CT, computed tomography; IV, intravenous

**Figure 2:**
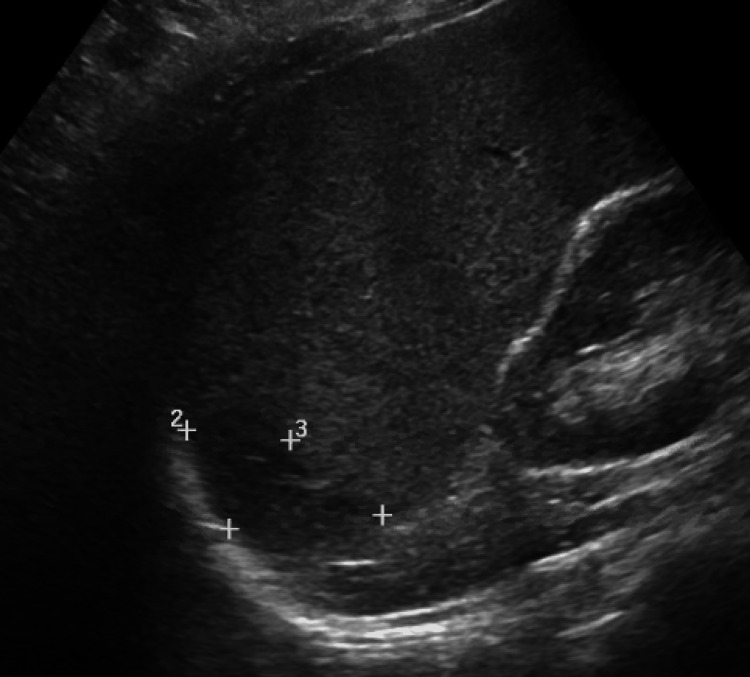
Abdominal ultrasound revealing splenomegaly and hypoechoic area measuring 5.4 cm × 3.7 cm × 3.7 cm.

An initial computer tomography (CT) angiogram of the chest was negative for pulmonary embolism. A CT of the abdomen and pelvis showed splenomegaly measuring 18 cm in length, with a wedge-shaped hypodensity at the posterior and medial aspect, consistent with an area of splenic infarction (Figure 1). An abdominal ultrasound noted splenomegaly with a small hypoechoic area in the spleen measuring 5.4 cm × 3.7 cm × 3.7 cm (Figure 2). A subsequent CT scan of the abdomen/pelvis two days later measured the spleen to be 17 cm, with a wedge-shaped, low-attenuation posterior superior infarct likely to be subacute to chronic.

Differential diagnoses for this patient’s splenic infarct included viral illness, acute hepatitis, inherited coagulopathy, blood-borne malignancy, myeloproliferative disease, autoimmune disease, pancreatitis, and thromboembolism. Further immunological, hematologic, and viral laboratory tests were ordered as his liver enzymes continued to rise, with AST reaching 260 IU/L, ALT 331 IU/L, and ALP 196 IU/L. The serological tests for CMV, HIV, and COVID-19 were all negative. The hepatitis panel was also negative. Hematology was consulted for further evaluation of the patient’s polycythemia. The patient denied exogenous hormone use has caused his polycythemia. Peripheral blood smear revealed no clumping, teardrop erythrocytes, or schistocytes. Antinuclear antibodies, antiphospholipid, lupus anticoagulant, anti-B2 glycoprotein I, and cardiolipin antibodies were undetected, ruling out autoimmune disease. Amylase and lipase levels were within normal range, ruling out pancreatitis. Atherosclerotic disease in the setting of obesity was unlikely, given an unremarkable lipid panel, and EKG was negative for atrial fibrillation. It was also considered that the patient’s diarrhea may have induced a hypovolemic state, causing demand ischemia leading to infarction. However, a positive Monospot test confirmed the diagnosis of EBV infection, with a positive EBV VCA-IgM antibody (>4.0 AI). EBV VCA-IgG, EA antibody (EA-D), and EBNA antibody were negative. It was concluded that the patient’s splenic infarct was caused by EBV-induced IM.

On the day of his discharge, the patient’s Monospot test remained positive, with AST 260 IU/L, ALT 331 IU/L, ALP 196 IU/L, and total bilirubin 1.7 mg/dL - all trending up from the date of admission. The patient was treated with intravenous fluids, analgesics, and therapeutic enoxaparin. He was discharged home in stable condition with anti-inflammatory medications and pain control and was placed on apixaban. He was recommended for immediate follow-up with Hematology Outpatient. Ten days later, a repeat abdominal ultrasound showed a continued moderately enlarged spleen (20 cm × 9 cm × 8 cm) with the persistence of the splenic infarct, measuring approximately 5 cm. Months later, a subsequent abdominal CT showed a spleen normal in size with no focal abnormalities.

## Discussion

IM is a clinical syndrome that presents as a triad of fever, pharyngitis, and lymphadenopathy, often accompanied by malaise, myalgias, and fatigue [1]. Splenic infarction is a rare complication of IM due to EBV infection. We performed a simple literature search for the terms “(Splenic Infarct)” and “(Infectious Mononucleosis OR Epstein-Barr Virus)” from 1990 to 2020, which resulted in 46 articles. Of these, 31 were found to detail 33 unique case reports of splenic infarctions in the setting of IM with heterophile antibody or serology results positive for EBV. While most of the cases in the literature presented with this triad or at least two of the three components, nine of the 33 cases presented with fever alone, as in our case. In only one case, the patient did not present with any symptoms of the classic triad and instead was found to have a splenic infarct [1]. In our literature search, six of the cases also reported negative heterophile antibody tests with positive EBV serological or DNA PCR tests. This demonstrates the importance of persistence in pursuing a diagnosis of suspected EBV infection with more sensitive testing when the Monospot test returns a negative result.

Both ultrasound and CT may be used to diagnose splenic infarction. Ultrasound is a reasonable first-line imaging, as this modality is inexpensive and can be performed easily and quickly at the bedside [9]. The *bright band sign* is a useful sonographic sign that can be found in this context, demonstrating thin, linear, parallel echogenic bands within the infarcted area in the spleen. However, ultrasound is not 100% sensitive. For example, one small retrospective study in 2009 showed that ultrasound was only diagnostic in 18% of splenic infarctions diagnosed by CT [7,10]. CT with contrast completed during the portal venous phase is typically considered the imaging modality of choice. The appearance of a splenic infarction on imaging largely depends on the timing of occurrence and size of the infarct. The standard CT finding indicative of a splenic infarct is a peripheral wedge-shaped area of hypodensity. In the hyperacute phase, there may be areas of increased attenuation that appear mottled, signifying areas of hemorrhagic infarction. In a global splenic infarction, the entire spleen exhibits hypoenhancement, such as with splenic torsion. If multiple infarcts are present, they may display several hypodense non-enhancing lesions among normal enhancing splenic tissue. In the chronic phase, the splenic infarcts may not appear at all, but instead, imaging may show a fibrotic contraction of the area of the infarct with hypertrophy of the adjacent healthy splenic tissue. If the infarct has begun to undergo liquefactive necrosis, imaging may demonstrate a cystic lesion with a central fluid density [11].

One retrospective study in 2009 that reviewed cases of acute splenic infarctions over 10 years found that three out of 48 cases were associated with EBV infections [7]. Thus, serology for EBV should be part of the standard workup for splenic infarction, even in patients who do not display the classic triad of symptoms. 

Reports of splenic infarctions in the setting of EBV-induced IM before 2010 were limited (only nine total) and mostly associated with patients with underlying hematologic comorbidities, such as sickle cell trait, and hereditary spherocytosis [12,13]. However, reports of this have increased in the last decade, with 24 being published since 2010. The rising number of reports of this complication of IM suggests that it might not be as rare as previously thought. Furthermore, most of these cases have occurred in previously healthy individuals, without underlying hematologic abnormalities. Only three out of 24 cases have occurred in patients with preexisting hematologic comorbidities since 2010, compared to four out of nine cases before 2010. Given this data, clinicians should maintain high suspicion of EBV-induced splenic infarction even in previously healthy individuals. 

Due to the limited number of reports of this condition and lack of extensive research, the pathophysiology behind splenic infarction in the setting of EBV infection remains a mystery. However, there are several reasonable explanations currently believed to play a role in this phenomenon. One hypothesis is demand ischemia in the setting of a rapidly enlarging spleen, as commonly occurs in IM. The proliferation of EBV-infected B-cells and reactive T-cells, production of various cytokines, and deposition of EBV-induced hemolyzed erythrocytes lead to acute expansion of red and white pulp. The hypercellularity of splenic cords during IM compacts the sinus structure and interrupts blood flow while increasing oxygen consumption, thereby creating demand ischemia [14]. Additionally, EBV-induced hemolysis can cause anemia, especially in the setting of underlying hematologic diseases such as sickle cell disease, sickle cell trait, hereditary spherocytosis, or pyruvate kinase deficiency, further contributing to hypoxia and increasing the risk of ischemia in these patients [13].

Furthermore, there is thought to be an increased thrombotic tendency in the acute phase of EBV infection that makes patients susceptible to splenic infarction. The proliferation of atypical B-cells can result in increased production of various antibodies. Guibaud et al. reported a case of IM-related splenic infarction in 1983, in which the patient was found to have high levels of serum immune complexes with resultant leukocyte aggregation, which they believed to contribute to arterial occlusion [15]. Additionally, numerous case reports have reported elevated levels of thrombophilic antibodies with positive tests for antiphospholipid antibodies, lupus anticoagulant, anti-cardiolipin, smooth muscle antibodies, or beta-2 glycoprotein I during the acute phase of IM [5,16,17-19]. The patient's family history of Evans syndrome in his mother could also be further investigated; if this patient also has this condition, it might offer another explanation for his SI. 

While there are likely other mechanisms contributing to this phenomenon, current research is limited, and they are difficult to ascertain at this time. Many case reports, including ours, did not perform a full workup for thrombotic abnormalities during the acute phase of the illness. As such, further research is required to determine the full pathophysiology underlying splenic infarction in EBV-induced IM.

There is no definitive or specific treatment for splenic infarction, as it is usually based on the underlying cause. In the absence of an infectious etiology, most treatments are supportive, involving rest, hydration, analgesics, antipyretics, antiemetics, and other supportive care measures, if necessary. Patients should also receive a thorough workup for underlying hemoglobinopathies or thrombophilia and to determine the need for anticoagulation. Consultation with hematology, oncology, or rheumatology may also be needed [20]. Splenic infarcts may sometimes become infected, leading to abscess formation, or undergo a hemorrhagic transformation, which requires emergent surgical evaluation. For this reason, splenic infarctions should be closely monitored. Patients should also be closely followed up outpatient following discharge, to monitor any abnormal lab findings during the acute phase of EBV infection to confirm their return to normal within a few months. Anticoagulation or antiplatelet therapy may be prudent if laboratory abnormalities persist to prevent a recurrence, although the risk of recurrence in such a situation in unknown [18]. The necessity of follow-up imaging is controversial but may be useful to verify the resolution of splenic lesions. One study recommends follow-up imaging after at least four weeks to determine improvement in splenic infarction associated with IM [14].

## Conclusions

While IM is a very common condition in younger patients, splenic infarction is a much more infrequent complication. Splenic infarction is a serious complication of IM, and it may be more common than previously thought. Young patients, especially those aged around 15-25 years and presenting with LUQ pain along with flu-like symptoms should be tested for EBV. Physicians should also consider other complications in addition to just splenomegaly, such as splenic infarction, even in patients who are otherwise healthy, nonsmoking, and without previous history of coagulopathy or hematologic abnormalities. EBV testing may also aid with the diagnosis of young patients found to have splenic infarction on imaging if the cause is unknown.
